# Prevalence and Characteristics of Obsessive-Compulsive Disorder Among Urban Residents in Wuhan During the Stage of Regular Control of Coronavirus Disease-19 Epidemic

**DOI:** 10.3389/fpsyt.2020.594167

**Published:** 2020-12-16

**Authors:** Yage Zheng, Ling Xiao, Yinping Xie, Huiling Wang, Gaohua Wang

**Affiliations:** Department of Psychiatry, Renmin Hospital of Wuhan University, Wuhan, China

**Keywords:** prevalence, distributions, risk factors, regular epidemic control and prevention, OCD, COVID-19

## Abstract

**Background:** Coronavirus disease-19 (Covid-19) is one of the most devastating epidemics in the 21st century, which has caused considerable damage to the physical and mental health of human beings. Despite a few regions like China having controlled the epidemic trends, most countries are still under siege of COVID-19. As the emphasis on cleaning and hygiene has been increasing, the problems related to obsessive-compulsive disorder (OCD) may appear.

**Objective:** This study was designed to investigate the prevalence of OCD in the urban population in Wuhan during the stage of regular epidemic control and prevention. Meanwhile, characteristics and risk factors for OCD were also explored.

**Method:** Five-hundred and seventy residents in urban areas of Wuhan were recruited using the snowball sampling method to complete questionnaires and an online interview from July 9 to July 19, 2020. Collected information encompassed socio-demographics, Yale-Brown Obsessive-Compulsive Scale (Y-BOCS) scores, Social Support Rating Scale (SSRS) scores and Pittsburgh Sleep Quality Index(PSQI) values.

**Results:** Three months after lifting the quarantine in Wuhan, the prevalence of OCD was 17.93%. About 89% of OCD patients had both obsessions and compulsions, while 8% had only obsessions and 3% had only compulsions. Top 3 common dimensions of obsessions were miscellaneous (84.0%), aggressive (76.6%), and contamination (48.9%), and of compulsions were miscellaneous (64%), checking (51.7%), and cleaning/washing/repeating (31.5%). The unmarried were more vulnerable to OCD than the married (*p* < 0.05, odds ration = 1.836). Students had 2.103 times the risk of developing OCD than health care workers (*p* < 0.05). Those with positive family history of OCD and other mental disorders (*p* < 0.05, odds ration = 2.497) and presence of psychiatric comorbidity (*p* < 0.05, odds ration = 4.213) were also at higher risk. Each level increase in sleep latency increased the risk of OCD to 1.646 times (*p* < 0.05).

**Conclusion:** In the background of regular epidemic control, the prevalence of OCD was high, and the symptoms were widely distributed. Obsessions often accompanied compulsions. Being single and a student, positive family history of OCD and other mental disorders, presence of psychiatric comorbidity, and longer sleep latency were predictors of OCD. Early recognition and detection of these issues may help to intervene in OCD.

## Introduction

The new coronavirus disease (COVID-19), which was first detected in December 2019, was declared as a public health emergency of international concern (PHEIC) on January 30, 2020 (1). Due to the rapid spread of the infection and paucity of available medical resources, the entire world was affected within a short time. The medical service system was once on the brink of collapse, facing the seemingly invincible “rival.” As of August 8, 2020, the total number of confirmed cases had approached 19,295,350, among whom, 719,805 people had lost their lives (2). As one of the first few countries which were heavily hit by the pandemic for a long time, mainland China has almost succeeded in managing the situation. Despite some slight increase in numbers of contingent and sporadic cases, Wuhan was reopened on April 8, 2020, and financial status and social businesses were gradually brought back on track. Notwithstanding, mental health seems to be a pending problem worth close attention.

Apart from causing serious damage to the human body, infectious diseases tend to influence mental health (3); the same is the case with COVID-19. Since the outbreak of this unprecedented pandemic, a swarm of studies across nations indicated an increased prevalence of mental disorders. For example, a study from China found that 40.4% of the local youth were mentally distressed, among whom ~1/3rd had symptoms of post-traumatic stress disorder (4). Another study of the adult population in Bangladesh found that 33.7% of the sample population was anxious, and 57.9% was depressed (5). However, previous studies were largely based on statistics at the beginning or peak of the pandemic, and no studies have investigated the mental status of the population in the later stage. After all, the quarantine has been lifted for months in Wuhan, China. According to an earlier review, the most focused mental disorders were anxiety, depression, post-traumatic stress disorder, stress, and not much attention was paid to obsessive compulsive disorder (OCD) (6).

OCD, mainly characterized by recurrent intrusive thoughts (obsessions) and repetitive stereotyped behaviors (compulsions), is a common chronic mental disease, which is often under-recognized (7). The estimated lifetime prevalence is usually believed to be 2–3% (8). As one of the top 10 diseases contributing to the Global Burdens of Disease, it is also related to suicide (9, 10). The fact that OCD could last for decades has also been mentioned in some clinical and community researches (11). Trauma, originally considered as a cause of post-traumatic stress disorder, could influence OCD to some degree (12, 13). A recent study conducted among OCD cases in Italy found a higher Y-BOCS score after 6 weeks of quarantine, indicating possible changes in OCD severity. However, studies rarely discussed the occurrence of OCD among the general population (14). After all, psychological reconstruction is an upcoming challenge. Is the prevalence of OCD still high in the epidemic stage?

Social support and sleep quality have been linked to mental health in many previous studies; enough social support and good sleep quality could ensure a better mood (15, 16). However, the association between them seems to be complicated. For example, family members are challenged in terms of offering support, which is helpful for patients with OCD, but to not let this support turn into family accommodation, which may lengthen the duration of OCD symptoms because anxiety is avoided in these patients (17). Jacob A. Nota found that delayed sleep phases were common in patients receiving intensive OCD treatment and later bedtimes were associated with more severe OCD symptoms both during admission and after discharge, however, no evidence revealed the same prediction for sleep onset latency or duration (18). No exploration on correlation between sleep quality and OCD in this special situation was found. Accordingly, more studies should be made to elucidate the relationship between social support or sleep quality and OCD in the later stage of the epidemic.

Currently, most countries are still under the siege of Covid-19. The occurrence of mental problems may be delayed, and these problems can persist for a long time. Therefore, the mental health effects of the pandemic need to be investigated. Would people in reopened areas suffer from OCD in the background of regular epidemic prevention and control?

Thereby, we investigated urban residents in Wuhan, aiming at collecting concrete clues on OCD and its risk factors, which might, in turn, assist in providing valuable reference for other countries as well as handling this issue instantaneously and potentially. The hypotheses for this study were the following.

Hypothesis 1: the prevalence of OCD in the regular epidemic stage is higher than what it used to be pre-pandemic.

Hypothesis 2: social support and sleep quality may help to predict OCD diagnosis in this background.

## Materials and Methods

### Participants

People from central areas of Wuhan, China, were recruited online through “Wenjuanxin” and “WeChat” using the snowball sampling method from July 9 to July 19, 2020, around 3 months since the quarantine had been lifted.

Inclusion criteria were: (1) a resident of a central urban area in Wuhan, (2) aged 15 years or above, and (3) ability to understand the contents of questionnaires.

People were excluded if they: (1) could not meet the inclusion criteria, (2) spent <2 min or over 1 h for filling questionnaires, (3) stumbled upon “trap questions,” or (4) dropped out.

Eleven participants spent over 1 h for filling questionnaires, 17 failed in “trap questions,” and 1 quit midway. Thus, 29 invalid questionnaires were eliminated, and 541 samples were included in the analysis; the valid response rate was 94.91%.

All respondents participated voluntarily under the premise of written informed consent and could quit at any time. Ethical approval was obtained from Renmin Hospital of Wuhan University.

### Measures

#### Demographics

Several socio-demographic characteristics, such as sex, age, income, marital status, educational level, and number of family members, were included in the questionnaire (Table 1).

**Table 1:** Socio-demographic characteristics of residents included in the study.

**Demographic variables**	**Classification**	***N***	**%**
Gender	Male	230	42.5
	Female	311	57.5
Age group (years)	15–24	128	23.7
	25–34	242	44.7
	35–44	99	18.3
	≥ 45	72	13.3
Monthly pay (RMB)^a^	<3 k	177	32.7
	3–5k	175	32.4
	> 5 k	189	34.9
Marital status	Unmarried	237	43.8
	Married	304	56.2
Education level	High school	114	21.1
	Junior college	123	22.7
	Bachelor	195	36.1
	Master degree	109	20.1
Number of family members	1	33	6.1
	2–3	299	55.3
	4–6	191	35.3
	> 6	18	3.3
Employment status	Employed	346	64.0
	Retired	25	4.6
	In school	120	22.2
	Unemployed	50	9.2
Occupation	HCWs^b^	144	26.6
	Students	113	20.9
	Others	284	52.5
District	Wuchang	228	42.1
	Qiaokou	30	5.5
	Jiangan	30	5.5
	Jianghan	55	10.2
	Hongshan	132	24.4
	Hanyang	44	8.1
	Qinshan	22	4.1
Duration of residence (months)^b^	0–6	101	18.7
	6–12	74	13.7
	12–36	88	16.3
	>36	278	51.4
Exposure level^c^	Low	444	82.1
	Medium	73	13.5
	High	24	4.4
Confirmed case^d^	Yes	12	2.2
	No	529	97.8
Suspected case^d^	Yes	15	2.8
	No	526	97.2
Asymptomatic case^d^	Yes	20	3.7
	No	521	96.3
Other mental disease	Yes	41	7.6
	No	500	92.4
Family history^e^	Yes	28	5.2
	No	513	94.8
SSRS score, mean ± SD	37.24 ±8.55		
Subjective support score, mean ± SD	22.20 ± 5.25		
Objective support score, mean ± SD	8 ± 3.37		
Availability of support score, mean ± SD	7.04 ± 2.06		
Modified PSQI score, mean ± SD	7.75 ± 2.79		
Sleep quality score, mean ± SD	1.97 ± 0.75		
Sleep disturbance score, mean ± SD	2.21 ± 1.08		
Sleep latency score, mean ± SD	1.80 ±1.00		
Sleep duration score, mean ± SD	1.77 ± 0.82		

a *RMB is China's currency, also know as yuan*;

b *duration of residence enquired as, “How long have you been in Wuhan?”*;

c *exposure levels of residents without contact with potentially infected people, residents who had contact with potential patients but were not in a continuous exposure to the virus, and those shuttling across hospitals or patients everyday (frequent contact with the virus) are considered low, middle, and high exposure levels, respectively*;

d *confirmed/suspected/asymptomatic refer to COVID-19 status*;

e *A family history refers but is not confined to OCD; other mental disorders like schizophrenia, depressive disorder, maniac disorder, bipolar disorder, anxiety disorders, post-traumatic stress disorder, Tourette syndrome are also included*.

*HCWs, health care workers; PSQI, Pittsburgh Sleep Quality Index; SSRS, Social Support Rating Scale; SD, standard deviation*.

In particular, information on family history of mental disorders or comorbid mental disorders was acquired by items in the questionnaire saying “Have you ever been diagnosed with a mental illness like schizophrenia, depressive disorder, maniac disorder, bipolar disorder, anxiety disorders, post-traumatic stress disorder, Tourette syndrome in the hospital and remained uncured,” “Do you have family members diagnosed with OCD or mental disorders like above?” We also reconfirmed this information orally in a brief online interview. Respondents who reported the presence of an additional mental disorder as well as the context where the diagnosis was given, plus the duration of the disorder, were confirmed as participants with psychiatric comorbidity. The same criteria were applied to ascertain a positive family history for a mental disorder.

#### Yale-Brown Obsessive-Compulsive Scale (Y-BOCS)

The widely used Y-BOCS consists of a checklist for symptoms (58 items) and a scale for severity (10 items, with each item scored from 0 to 4, and a total point ranging from 0 to 40). There is a moderate correlation in consistency and discrepancy between self-reported and clinician-rated Y-BOCS scores and patients tend to rate symptoms lower than clinicians from experience (19, 20). Given its availability in self-report format, Y-BOCS was applied for the assessment of diagnosis and manifestations of OCD as an online questionnaire and interview. A cut-off point of 6 was considered for the diagnosis of OCD (21).

A 10-min oral online interview was conducted for all participants through the “Wechat” app (a worldwide communication application, similar to Facebook, Skype, etc.) for rendering explanations of purpose of this research, reconfirmation of participation, as well as interpretation of colloquial definition of obsessions and compulsions, thereby aiming to minimize the confusion to a maximum level.

#### Social Support Rating Scale (SSRS)

The Chinese version of SSRS designed by Shuiyuan Xiao was used to evaluate the type and levels of social support received from others. The questionnaire consists of 3 aspects, namely, subjective social support, objective social support, and the availability of social support, with a total point ranging from 7 to 56. The more the points you score, the more the social support you have (22).

#### Pittsburgh Sleep Quality Index (PSQI)

The modified PSQI with 4 dimensions, such as sleep satisfaction, sleep disturbance, sleep latency, and sleep duration, was applied to appraise sleep quality. Each dimension is scored between 0 and 3, with a total point ranging from 0 to 21, and the more the points you score, the poorer the quality of your sleep (23).

#### Statistical Analysis and Data Processing

SPSS 24.0 software (Armonk, NY: IBM Corp) was used for statistical analyses. The dependent variable in the current study, OCD or non-OCD, was categorical, while independent variables consisted of both categorical and quantitative ones. Thereby, comparisons of group differences in categorical data were performed by the chi square test. Quantitative variables with normal distribution were processed using the *T*-test, while non-normally distributed variables were processed using non-parametric tests. A *p* < 0.05 indicated a significant difference. Besides, all significant factors in univariate analysis as well as those believed to be relevant variables were introduced in a multi-factorial logistic regression stepwise equation (LR, Forward) for a deeper insight into relatively independent risk factors of OCD; *p* < 0.05 indicated significance.

## Results

### Description of Samples

Five-hundred and seventy residents from all 7 central urban areas in Wuhan participated in the research, 29 among whom were excluded due to invalid response to the questionnaire. Thus, 541 appropriate respondents were included; 57.5% of them were females, and the rest were males. Most respondents were young (86.7%). About 7.6% of respondents admitted to having co-morbidity with other mental diseases, and 5.2% claimed to have family members with mental disorders. Complete baseline information is shown in Table 1.

### Distribution of OCD Symptoms

In total, 97 respondents were confirmed to have OCD according to Y-BOCS, among whom 86 had both obsessions and compulsions; obsessions (*n* = 8) or compulsions (*n* = 3) presenting alone were rare.

For a clearer understanding of the manifestations of symptoms, the Y-BOCS symptom checklist was introduced. As shown in Table 2, a wide range of distribution of manifestations of obsessions and compulsions was observed. Top 3 obsessions were miscellaneous (84.0%), aggressive (76.6%), and contamination (48.9%); top 3 compulsions were miscellaneous (64%), checking (51.7%), and cleaning/washing/repeating (31.5%).

**Table 2:** The detailed distribution of symptomatic dimensions among people with OCD.

	**Obsessions**	**N (%)**	**Compulsions**	**N (%)**
Dimensions	Aggressive	72 (76.6)	Checking	46 (51.7)
	Contamination	46 (48.9)	Cleaning/washing	28 (31.5)
	Hoarding/saving	25 (26.6)	Hoarding/collecting	16 (18.0)
	Symmetry or exactness	22 (23.4)	Ordering/arranging	24 (27.0)
	Miscellaneous	79 (84.0)	Miscellaneous	57 (64.0)
	Sexual	24 (25.5)	Repeating	28 (31.5)
	Religious	27 (28.7)	Counting	6 (6.7)
	Somatic	39 (41.5)		

### Group Differences of OCD in Socio-Demographics, Social Support, and Sleep Quality

Altogether, 97 respondents met the criterion for OCD diagnosis, so the prevalence of OCD in the background of regular epidemic prevention and control was 17.93%. The prevalence of OCD increased as age decreased, with the highest being 22.66% in the young group aged between 15 and 24 years (*p* < 0.05). Further, the univariate analysis indicated that the prevalence of OCD diagnosis differed depending on some sociodemographic variables such as marital status, occupation, and employment status (*p* < 0.05). Moreover, the prevalence in respondents who were asymptomatic cases, with comorbid mental disorders, family history of OCD or other mental disorders, sleep disorders, or poor social support levels turned out higher than that in those without these factors (*p* < 0.05).

### Predictors for OCD

Significant variables from Supplementary Table 1 and those non-significant but believed to be relevant factors from past experience (gender, education level) (24, 25) were all included in the multivariate logistic regression model; finally, as listed in Table 3, several variables were identified as predictors for OCD. Compared to the married, the respondents who were single were at 1.836 times the risk of having OCD (*p* < 0.05). Students were at 2.169 times the risk of having an OCD diagnosis compared to that of health care workers (HCWs). The prevalence of OCD in people with comorbid mental disorders or a positive family history of OCD or other mental disorders was much higher than that in those without other mental disorders (*p* < 0.05). Notably, sleep latency, which was one of the assessments for sleep quality in the current research, turned out to be an independent predictor for OCD; each unit increase in sleep latency was associated with 0.646 times higher risk for developing OCD (*p* < 0.05).

**Table 3:** Multi-factorial stepwise logistic regression analysis of related factors.

**Variable**	**B**	**SE**	**Wald**	**df**	**Sig**	**Exp(B)**	**95% CI (lower-upper)**
Single	0.608	0.279	4.728	1	0.030	1.836	1.062–3.175
HCWs			6.350	2	0.042		
Students	0.774	0.357	4.693	1	0.030	2.169	1.077–4.370
Others	0.031	0.311	0.010	1	0.921	1.031	0.561–1.897
Comorbidity	1.438	0.377	14.520	1	0.000	4.213	2.011–8.828
Family history	0.915	0.462	3.924	1	0.048	2.497	1.010–6.176
Sleep latency	0.499	0.115	18.803	1	0.000	1.646	1.314–2.063

## Discussion

To the best of our knowledge, this is the first study on the prevalence of OCD and possible influencing factors among central urban residents in Wuhan in the background of regular epidemic control and prevention. As known to all of us, Wuhan, one of the first areas that were heavily thrashed by COVID-19, has achieved great success in the battle against this pandemic through hard work and generous support from all circles. New cases have not been observed since March 18, 2020, and the lockdown policy was removed on April 8, 2020, under the premise of the mitigated situation.

Notwithstanding, something worth much attention is the fact that people from this area might still suffer from certain mental disorders in the stage of regular epidemic prevention. As observed in this study, despite being a relatively secure area compared with many other countries where the pandemic progressed, 3 months after reopening, people in Wuhan were still affected by OCD with a prevalence rate of 17.93%. Occupation, marital status, comorbid mental disorders, family history and sleep latency were associated with OCD.

To date, very limited studies have focused on OCD. An earlier study with Symptom checklist-90 indicated that the prevalence rates of OCD symptoms among HCWs and non-HCWs were 5.3 and 2.2%, respectively (26). Similar to other mental diseases, OCD was pervasive among participants in our study, with prevalence rates of 14.6% for HCWs, 29.2% for students, and 15.1% for others. Students had 2.169 times the risk of developing OCD compared with that of HCWs (*p* < 0.05), indicating that students were the vulnerable ones. Indeed, students, under great pressure and with dubiously-oriented coping skills were often prone to mental disorders (27). Hence, more attention from the education sector is warranted. It is also important to note that we did not classify students into more detailed categories according to majors (e.g., medicine, art or music, computer or Internet) or grades (e.g., freshman, sophomore, junior, senior). Hence, it would be too early to figure out whether differences exist between medical students and HCWs or across subgroups. Further research in this direction would help to address this problem. Marriage was another predictor of OCD; the risk of developing OCD in the unmarried population was 1.836 times greater than that in the married population. Previous studies have shown that marital status contributed meaningfully to the quality of life; meanwhile, nearly all domains of quality of life seemed to have degenerated in patients with OCD (28–30). This may be the reason that marriage acted as a predictor of OCD in our study. Comorbid status is typical of OCD, as indicated in one study, where in ~80% of cases, OCD occurred at a certain stage after being diagnosed with anxiety (31). In our research, people with other concurrent mental disorders were more prone to develop OCD (48.8 vs. 15.4%, *p* < 0.05, odds ration = 4.213). A family history of OCD also increased the risk of developing OCD with an odds ratio of 2.497. Previously, both twin studies and genome-wide association reports indicated the heritability of OCD (32, 33). Regarding sleep quality, difficulty in falling asleep was a predictor of OCD. The risk of developing OCD seemed to increase by 64.6% for every increase in the level of sleep latency. People were reported to have many sleep problems during the quarantine, which may have been associated with the risk of developing OCD (15, 16). Consistent with this, in our study, many patients with OCD had disturbing thoughts and repetitive performances like counting or making the bed, which in turn influenced the development of OCD. Several research articles and meta-analysis found that depression and anxiety play a key role in the sleep disturbances among OCD patients (34–36). Unfortunately, we did not recruit subjects with anxiety and depressive disorders, but an emerging idea of interactive effects among these mental diseases should be explored in future investigation. There are insufficient investigations focused on the distribution of obsessions and compulsions. A study on Chinese Han population in 2012 indicated that the commonly detected obsessions were aggressive (42.4%), miscellaneous (42.2%), and contamination (21.6%); while compulsions were checking (52.1%), miscellaneous (25.2%), and washing/cleaning (25.2%) (37). Compared with their results, our study showed a more wide-ranging distribution of overlapped symptoms, and detection rates of both obsessions and compulsions related to hygiene in our study were higher than in theirs. The exact influencing factors are opaque at present, but, as pointed out, a distinction does exist regarding the distribution and detection rate of symptoms. Medical response refers to how we react to the pandemic medically, what measures we prefer to take to contain the pandemic, the speed with which we take medical-related actions, etc. Further research should clarify if the symptom dimension/severity would be associated with the ways people deal with the COVID-19. In our study, some variables such as age, employment status, asymptomatic status, and social support, which were significant factors in univariate analysis, revealed no significance in the multi-factorial regression model. This could be explained by interactions between the variables and differences in research periods, populations, or selected scales. The local government did provide much support for people in Wuhan, such as providing coupons and tax deduction; therefore, the fact that basic level of social support was high might be another reason that this variable was not a significant factor. However, our study found a somewhat high prevalence of OCD even in the regular pandemic control stage, which might provide some basic information or reference for other countries.

### Limitations and Prospects

Despite several findings mentioned above, our study has some limitations. First, the cross-sectional design with a limited sample size makes it hard to figure out a causal relationship between the factors and OCD; therefore, in future research, we will be following-up on these residents as well as including a larger sample as possible. Second, considering the fact that people experienced a huge impact not so long ago, a more unbiased randomized sampling method was not applied; it should be adopted in future research when appropriate. Third, we did not compare the differences among groups with different levels of OCD (mild, moderate, severe); future research should address this limitation. Finally, it remains to be seen to what extent and how people from other parts of the world experience OCD in this special situation.

### Conclusions

The present cross-sectional study conducted among urban people in Wuhan indicated that OCD, with wide-ranging symptomatic dimensions, was very pervasive in the stage of regular epidemic control. In addition, it was observed that obsessions and compulsions occurred independently. Being single or a student, family history of OCD, comorbid status, and longer sleep latency appear to be potential predictors of OCD in this situation; therefore, more attention should be paid to these factors, allowing for early detection and intervention in OCD.

## Data Availability Statement

The raw data supporting the conclusions of this article will be made available by the authors, without undue reservation.

## Ethics Statement

The studies involving human participants were reviewed and approved by the Clinical Research Ethics Committee of Renmin Hospital of Wuhan University. Written informed consent to participate in this study was provided by the participants' legal guardian/next of kin.

## Author Contributions

YZ, LX, and HW: design and concept. GW: supervision and management. YZ and LX: draft of manuscript. YZ and YX: processing, analysis of statistics, collection, acquisition, and verification of data. All authors contributed to the article and approved the submitted version.

## Conflict of Interest

The authors declare that the research was conducted in the absence of any commercial or financial relationships that could be construed as a potential conflict of interest.
