# Supplementary material for: Prevalence and Characteristics of Obsessive-Compulsive Disorder Among Urban Residents in Wuhan During the Stage of Regular Control of Coronavirus Disease-19 Epidemic
Source: Front Psychiatry. 2020 Dec 16;11:594167. doi: 10.3389/fpsyt.2020.594167 (PMC7772465; doi:10.3389/fpsyt.2020.594167)
Supplement: Supplementary file 1 [file Data_Sheet_1.pdf]

Name: ______________________________________ Date: ____________________

YBOCS

While completing the inventories below, please keep in mind the following definitions of obsessions and compulsions.

OBSESSIONS are unwelcomed and distressing ideas, thoughts, or impulses that repeatedly enter your mind. They may seem to occur against your will. They may be repugnant to you, you may recognize them as senseless, and they may not fit your personality.

Examples of an obsession are recurrent thought or impulses to do harm to a child even though you never would and the idea that household cleansers may lead to contamination and serious illness.

Obsessions differ from worries in that worries are about possible negative things related to life problems that you are afraid might happen. For example, you may worry about failing an exam, about finances, health, or personal relationships. In contrast to obsessions, your worries don't usually seem totally senseless, repugnant, or inconsistent with your personality.

COMPULSIONS, on the other hand, are behaviors or acts that you feel driven to perform although you may recognize them as senseless or excessive. Usually compulsions are performed in response to an obsession, or according to certain rules or in a stereotyped fashion. At times, you may try to resist doing them but this may prove difficult. You may experience discomfort that does not diminish until the behavior is completed.

Examples of a compulsions are the need to repeatedly check appliances, water faucets, and the lock on the front door before you can leave the house and repeated handwashing. While most compulsions are observable behaviors, some are unobservable mental acts, such as silent checking or having to recite nonsense phrases to yourself each time you have a bad thought.

Compulsions, as we define them here, are not to be confused with other kinds of compulsive behavior such as overeating, gambling, drinking alcohol, overshopping, or other "addictive behaviors."

Give the above definitions, please read carefully each item on the checklist below and 1) place a check mark beside each obsession and compulsion that you currently experience and that you have experienced at some time in the past. If you placed a check mark beside obsessions or compulsions that you currently experience; 2) circle the 2 most upsetting obsessions that you currently experience; and 3) circle the 2 most upsetting compulsions that you are currently engaged in.

YALE BROWN OBSESSIVE-COMPULSIVE SCALE SYMPTOM CHECKLIST

(GOODMAN, RASMUSSEN, ET AL.)

OBSESSIONS:

Aggressive Obsessions:

Past	Current		Examples
______	______	1. I fear I might harm myself	Fear of eating with a knife or fork, fear of handling sharp objects, fear of walking near glass windows
______	______	2. I fear I might harm other people	Fear of poisoning other people's food, fear of harming babies, fear of pushing someone in front of a train, fear of hurting someone's feelings, fear of being responsible by not providing assistance for some imagined catastrophe, fear of causing harm by giving bad advice.
______	______	3. I have violent or horrific images in my mind	Images of murder, dismembered bodies, or other disgusting scenes
______	______	4. I fear I will blurt out obscenities in class	Fear of shouting obscenities in public situations like church, fear of writing obscenities
______	______	5. I fear doing something embarrassing	Fear of appearing foolish in social situations
______	______	6. I fear I will act on an unwanted impulse	Fear of driving a car into a tree, fear of running someone over, fear of stabbing a friend
______	______	7. I fear I will steal things	Fear of "cheating" a cashier, fear of shoplifting inexpensive items
______	______	8. I fear that I'll harm others because I'm not careful enough	Fear of causing an accident without being aware of it (such as a hit-and-run accident)
______	______	9. I fear I'll be responsible for something else terrible happening.	Fear of causing a fire or burglary because of not being careful enough in checking the house before leaving

Contamination Obsessions:

Past	Current		Examples
______	______	10. I am concerned or disgusted with bodily waste or secretions	Fear of contracting AIDS, cancer, or other diseases from public rest rooms; fear of your own saliva, urine, feces, semen, or vaginal secretions
______	______	11. I am concerned with dirt or germs	Fear of picking up germs from sitting in certain chairs, shaking hands, or touching door handles

Past	Current		Examples
______	______	12. I am excessively concerned with environmental contaminants	Fear of being contaminated by asbestos or radon, fear of radioactive substances, fear of things associated with towns containing toxic waste sights
______	______	13. I am excessively concerned with certain household cleansers	Fear of poisonous kitchen or bathroom cleansers, solvents, insect spray or turpentine
______	______	14. I am excessively concerned with animals	Fear of being contaminated by touching an insect, dog, cat, or other animal
______	______	15. I am bothered by sticky substances or residues	Fear of adhesive tape or other sticky substances that may trap contaminants
______	______	16. I am concerned that I will get ill because of contamination	Fear of getting ill as a direct result of being contaminated (beliefs vary about how long the disease will take to appear)
______	______	17. I am concerned that I will contaminate others	Fear of touching other people or preparing their food after you touch poisonous substances (like gasoline) or after you touch your own body

Sexual Obsessions:

Past	Current		Examples
______	______	18. I have forbidden or perverse sexual thoughts, images, or impulses	Unwanted sexual thoughts about strangers, family, or friends
______	______	19. I have sexual obsessions that involve children or incest	Unwanted thoughts about sexually molesting either your own children or other children
______	______	20. I have obsessions about homosexuality	Worries like "Am I a homosexual?" or "What if I suddenly become gay?" when there is no basis for these thoughts
______	______	21. I have obsessions about aggressive sexual behavior toward other people	Unwanted images of violent sexual behavior toward adult strangers, friends, or family members

Hoarding/Saving Obsessions:

Past	Current		Examples
______	______	22. I have obsessions about hoarding or saving things	Worries about throwing away seemingly unimportant things that you might need in the future, urges to pick up and collect useless things

Religious Obsessions:

Past	Current		Examples
______	______	23. I am concerned with sacrilege and blasphemy	Worries about having blasphemous thoughts, saying blasphemous things, or being punished for such things
______	______	24. I am excessively concerned with morality	Worries about always doing “the right thing,” having told a lie, or having cheated someone

Obsession with need for Symmetry or Exactness:

Past	Current		Examples
______	______	25. I have obsessions about symmetry or exactness	Worries about papers and books being properly aligned, worries about calculations or handwriting being perfect

Miscellaneous Obsessions:

Past	Current		Examples
______	______	26. I feel that I need to know or remember certain things	Belief that you need to remember insignificant things like license plate numbers, the names of actors on television shows, old telephone numbers, bumper stickers or t-shirt slogans
______	______	27. I fear saying certain things	Fear of saying certain words (such as “thirteen”) because of superstitions, fear of saying something that might be disrespectful to a dead person, fear of using words with an apostrophe (because this denotes possession)
______	______	28. I fear not saying just the right thing	Fear of having said the wrong thing, fear of not using the “perfect” word
______	______	29. I fear losing things	Worries about losing a wallet or other unimportant objects, like a scrap of note paper
______	______	30. I am bothered by intrusive (neutral) mental images	Random, unwanted images in your mind
______	______	31. I am bothered by intrusive mental nonsense sounds, words or music	Words, songs, or music in your mind that you can’t stop
______	______	32. I am bothered by certain sounds or noises	Worries about the sounds of clocks ticking loudly or voices in another room that may interfere with sleeping

Past	Current		Examples
______	______	33. I have lucky and unlucky numbers	Worries about common numbers (like thirteen) that may cause you to perform activities a certain number of times or to postpone an action until a certain lucky hour of the day
______	______	34. Certain colors have special significance to me	Fear of using objects of certain colors (e.g. black may be associated with death, red with blood or injury)
______	______	35. I have superstitious fears	Fear of passing a cemetery, hearse, or black cat; fear of omens associated with death

Somatic Obsessions:

Past	Current		Examples
______	______	36. I am concerned with illness or disease	Worries that you have an illness like cancer, heart disease or AIDS, despite reassurance from doctors that you do not
______	______	37. I am excessively concerned with a part of my body or an aspect of my appearance (dysmorphophobia)	Worries that your face, ears, nose, eyes, or another part of your body is hideous, ugly, despite reassurances to the contrary

COMPULSIONS:

Cleaning/Washing Compulsions:

Past	Current		Examples
______	______	38. I wash my hands excessively or in a ritualized way	Washing your hands many times a day or for long periods of time after touching, or thinking that you have touched a contaminated object. This may include washing the entire length of your arms
______	______	39. I have excessive or ritualized showering, bathing, tooth brushing, grooming, or toilet routines	Taking showers or baths or performing other bathroom routines that may last for several hours. If the sequence is interrupted, the entire process may have to be restarted
______	______	40. I have compulsions that involve cleaning household items or other inanimate objects	Excessive cleaning of faucets, toilets, floors, kitchen counters, or kitchen utensils
______	______	41. I do other things to prevent or remove contact with contaminants	Asking family members to handle or remove insecticides, garbage, gasoline cans, raw meat, paints, varnish, drugs in the medicine cabinet, or kitty litter. If you can't avoid these things, you may wear gloves to handle them, such as when using a self-service gas pump

Checking Compulsions:

Past	Current		Examples
______	______	42. I check that I did not harm others	Checking that you haven't hurt someone without knowing it. You may ask others for reassurance or telephone to make sure that everything is all right
______	______	43. I check that I did not harm myself	Looking for injuries of bleeding after handling sharp or breakable objects. You may frequently go to doctors to ask for reassurance that you haven't hurt yourself
______	______	44. I check that nothing terrible happened	Searching the newspaper or listening to the radio or television for news about some catastrophe that you believe you caused. You may also ask people for reassurance that you didn't cause an accident
______	______	45. I check that I did not make a mistake	Repeated checking of door locks, stoves, electrical outlets, before leaving home; repeated checking while reading, writing, or doing simple calculations to make sure that you didn't make a mistake (you can't be certain that you didn't)
______	______	46. I check some aspect of my physical condition tied to my obsessions about my body	Seeking reassurance from friends or doctors that you aren't having a heart attack or getting cancer; repeatedly taking pulse, blood pressure, or temperature; checking your appearance in a mirror, looking for ugly features

Repeating Rituals:

Past	Current		Examples
______	______	47. I reread or rewrite things	Taking hours to read a few pages in a book or to write a short letter because you get caught in a cycle of reading and rereading; worrying that you didn't understand something you just read; searching for a "perfect" word or phrase; having obsessive thoughts about the shape of certain printed letters in a book
______________		48. I need to repeat routine activities	Repeating activities like turning appliances on and off, combing your hair, going in and out of a doorway, or looking in a particular direction; not feeling comfortable unless you do these things the "right" number of times

Counting Compulsions:

Past	Current		Examples
______	______	49. I have counting compulsions	Counting objects like ceiling or floor tiles, books in a bookcase, nails in a wall, or even grains of sand on a beach; counting when you repeat certain activities, like washing

Ordering/Arranging Compulsions:

Past	Current		Examples
______	______	50. I have ordering or arranging compulsions	Straightening paper and pens on a desktop or books in a bookcase, wasting hours arranging things in your house in "order" and then becoming very upset if this order is disturbed

Hoarding/Collecting Compulsions:

Past	Current		Examples
______	______	51. I have compulsions to hoard or collect things	Saving old newspapers, notes, cans, paper towels, wrappers and empty bottles for fear that if you throw them away you may need them; picking up useless objects from the street or from garbage cans

Miscellaneous Compulsions:

Past	Current		Examples
______	______	52. I have mental rituals (other than checking/counting)	Performing rituals in your head, like saying prayers or thinking a "good" thought to undo a "bad" thought. These are different from obsessions, because you perform them intentionally to reduce anxiety or feel better
______	______	53. I need to tell, ask, or confess	Asking other people to reassure you, confessing to wrong behaviors you never even did, believing that you have to tell other people certain words to feel better
______	______	54. I need to touch, tap, or rub things	Giving in to the urge to touch rough surfaces, like wood, or hot surfaces, like a stove top; giving in to the urge to lightly touch other people; believing you need to touch an object like a telephone to prevent an illness in your family

Past	Current		Examples
______	______	55. I take measures (other than checking) to prevent harm or terrible consequences to myself or family	Staying away from sharp or breakable objects, such as knives, scissors, and fragile glass
______	______	56. I have ritualized eating behaviors	Arranging your food, knife, and fork in a particular order before being able to eat, eating according to a strict ritual, not being able to eat until the hands of a clock point exactly at a certain time
______	______	57. I have superstitious behaviors	Not taking a bus or train if its number contains an "unlucky" number (like thirteen), staying in your house on the thirteenth of the month, throwing away clothes you wore while passing a funeral home or cemetery
______	______	58. I pull my hair out (trichotillomania)	Pulling hair from your scalp, eyelids, eyelashes, or pubic areas, using your fingers or tweezers. You may produce bad spots that require you to wear a wig, or you may pluck your eyebrows or eyelids smooth

Acknowledgments: The Y-BOCS was developed by Goodman, W.K., Price, L.H., Rassmussen, S.A., et al. (1989). The Yale-Brown Obsessive-Compulsive Scale (Y-BOCS) Part 1: Development, use and reliability. Archives of General Psychiatry,46 1006-1011. It was modified for computer administration by John Greist and associates, (1992). A computer administered version of the Yale-Brown Obsessive Compulsive Scale. Psychological Assessment, 4 329-332. The self-report version contained herein was developed by Lee Baer (1991).Getting Control: Overcoming your obsessions and compulsions. Boston: Little, Brown, & Co. The Y-BOCS Symptom Checklist was also developed by Dr. Wayne Goodman and associates. We extend our appreciation to Dr. Goodman and Dr. Baer for granting us permission to use these materials for clinical and research purposes

YALE BROWN OBSESSIVE-COMPULSIVE SCALE (Y-BOCS) – Part 2

Thank you for completing the Y-BOCS checklist. Please make sure you circle the 2 most upsetting obsessions that you currently experience and that you circled the 2 compulsions that cause you the most difficulty. Remember the definitions of obsessions and compulsions and the examples of each that you may have noted on the checklist. Please place a check mark by the appropriate number from 0-4 under each question below. If you are currently not experiencing any obsession or compulsions, you may simply enter zeros for the questions, then continue to the next question.

OBSESSIVE THOUGHTS: Review the obsessions you checked on the Y-BOCS Symptom Checklist to help you answer the first five questions. Please think about the <u>last seven days</u> (including today), and check one answer for each question.

1. TIME OCCUPIED BY OBSESSIVE THOUGHTS: How much of your time was occupied by obsessive thoughts? How frequently did these thoughts occur?

_____ 0 = None

_____ 1 = Less than 1 hour per day, or occasional intrusions (occur no more than 8 times a day)

_____ 2 = 1-3 hours per day, or frequent intrusions (most of the day are free of obsessions)

_____ 3 = More than 3 hours and up to 8 hours per day, or very frequent intrusions

_____ 4 = More than 8 hours per day, or near-constant intrusions

2. INTERFERENCE DUE TO OBSESSIVE THOUGHTS: How much did these thoughts interfere with your social or work functioning? Is there anything that you didn't do because of them?

_____ 0 = No interference

_____ 1 = Mild, slight interference with social or occupational performance, but still performance not impaired

_____ 2 = Moderate, definitive interference with social or occupational performance, but still manageable

_____ 3 = Severe interference, causes substantial impairment in social or occupational performance

_____ 4 = Extreme, incapacitating interference

3. DISTRESS ASSOCIATED WITH OBSESSIVE THOUGHTS: How much distress did your obsessive thoughts cause you?

_____ 0 = None

_____ 1 = Mild, infrequent, and not too disturbing distress

_____ 2 = Moderate, frequent, and disturbing distress, but still manageable

_____ 3 = Severe, very frequent, and very disturbing distress

_____ 4 = Extreme, near-constant, and disabling distress

4. RESISTANCE AGAINST OBSESSIONS: How much effort did you make to resist the obsessive thought? How often did you try to disregard or turn your attention away from those thoughts as they entered your mind?

_____ 0 = I made an effort to always resist (or the obsessions are so minimal that there is no need to actively resist them)

_____ 1 = I tried to resist most of the time (e.g. more than half the time I tried to resist)

_____ 2 = I made some effort to resist

_____ 3 = I allowed all obsessions to fill my mind without attempting to control them, but I did so with some reluctance

_____ 4 = I completely and willingly gave in to all obsessions

5. DEGREES OF CONTROL OVER OBSESSIVE THOUGHTS: How much control did you have over your obsessive thoughts? How successful were you in stopping or diverting your obsessive thinking?

_____ 0 = Complete control

_____ 1 = Much control; usually I could stop or divert obsessions with some effort and concentration

_____ 2 = Moderate control; sometimes I could stop or divert obsessions

_____ 3 = Little control; I was rarely successful in stopping obsessions and could only divert attention with great difficulty

_____ 4 = No control; I was rarely able to even momentarily ignore the obsessions

OBSESSION SUPTOTAL (Add items 1-5) ________

COMPULSIONS: Review the compulsions you checked on the Y-BOCS Symptom Checklist to help you answer these five questions. Please think about the <u>last seven days</u> (including today), and check one answer for each question.

6.TIME SPENT PERFORMING COMPULSIVE BEHAVIORS: How much time did you spend performing compulsive behavior? How frequently did you perform compulsions?

_____ 0 = None

_____ 1 = Less than 1 hour per day was spent performing compulsions, or occasional performance of compulsive behaviors (no more than 8 times per day)

_____ 2 = 1-3 hours per day was spent performing compulsions, or frequent performance of compulsive behaviors (most hours were free of compulsions)

_____ 3 = More than 3 hours and up to 8 hours per day were spent performing compulsions, or very frequent performance of compulsive behaviors (during most hours of the day)

_____ 4 = More than 8 hours were spent performing compulsions, or near-constant performance of compulsive behaviors (hour rarely passes without several compulsions being performed)

7. INTERFERENCE DUE TO COMPULSIVE BEHAVIOR: How much did your compulsive behaviors interfere with your social or work functioning?

_____ 0 = No interference

_____ 1 = Mild, slight interference with social or occupational activities, but overall performance not impaired

_____ 2 = Moderate, definite interference with social or occupational performance, but still manageable

_____ 3 = Severe interference, substantial impairment in social or occupational performance

_____ 4 = Extreme, incapacitation interference

8. DISTRESS ASSOICATED WITH COMPULSIVE BEHAVIOR: How would you have felt if prevented from performing your compulsion(s)? How anxious would you have become?

_____ 0 = Not at all anxious

_____ 1 = Only slightly anxious if compulsions prevented

_____ 2 = Anxiety would mount but remain manageable if compulsions prevented

_____ 3 = Prominent and very disturbing increase in anxiety if compulsions interrupted

_____ 4 = Extreme, incapacitating anxiety from any intervention aimed at reducing the compulsions

9. RESISTANCE: How much effort did you make to resist the compulsions? Or how often did you try to stop the compulsions?

_____ 0 = I made effort to always resist (or the symptoms were so minimal that there was no need to actively resist them)

_____ 1 = I tried to resist most of the time (e.g. more than half the time)

_____ 2 = I made some effort to resist

_____ 3 = I yielded to almost all compulsions without attempting to control them, but I did so with some reluctance

_____ 4 = I completely and willingly yielded to all compulsions

10. DEGREES OF CONTROL OVER COMPULSIVE BEHAVIOR: How much control did you have over the compulsive behavior? How successful were you in stopping the ritual(s)?

_____ 0 = I had complete control

_____ 1 = Usually I could stop compulsions or rituals with some effort and willpower

_____ 2 = Sometimes I could stop compulsive behavior but only with difficulty

_____ 3 = I could only delay the compulsive behavior, but eventually it had to be carried out to completion

_____ 4 = I was rarely able to even momentarily delay performing the compulsive behavior

COMPULSIVE SUBTOTAL (Add items 6-10) ____________
