# Supplementary material for: Prevalence and Characteristics of Obsessive-Compulsive Disorder Among Urban Residents in Wuhan During the Stage of Regular Control of Coronavirus Disease-19 Epidemic
Source: Front Psychiatry. 2020 Dec 16;11:594167. doi: 10.3389/fpsyt.2020.594167 (PMC7772465; doi:10.3389/fpsyt.2020.594167)
Supplement: Supplementary file 2 [file Data_Sheet_2.pdf]

Complete steps of multi-variable regression analysis

| | Variable | B | SE | Wald | df | Sig | Exp(B) | 95% CI (lower-upper) |
|---|---|---|---|---|---|---|---|---|
| Step1 | Sleep latency | 0.553 | 0.105 | 27.761 | 1 | 0.000 | 1.738 | 1.415-2.135 |
| | Constant | -2.612 | 0.253 | 106.951 | 1 | 0.000 | 0.073 | |
| Step2 | Comorbidity | 1.418 | 0.352 | 16.263 | 1 | 0.000 | 4.129 | 2.073-8.226 |
| | Sleep latency | 0.492 | 0.108 | 20.667 | 1 | 0.000 | 1.636 | 1.323-2.022 |
| | Constant | -2.643 | 0.257 | 105.741 | 1 | 0.000 | 0.071 | |
| Step 3 | HCWs | | | 16.454 | 2 | 0.000 | | |
| | Students | 1.095 | 0.332 | 10.918 | 1 | 0.001 | 2.991 | 1.562-5.728 |
| | Others | 0.024 | 0.305 | 0.006 | 1 | 0.937 | 1.024 | 0.563-1.863 |
| | Comorbidity | 1.574 | 0.367 | 18.410 | 1 | 0.000 | 4.825 | 2.351-9.901 |
| | Sleep latency | 0.519 | 0.111 | 21.687 | 1 | 0.000 | 1.680 | 1.350-2.090 |
| | Constant | -3.006 | 0.355 | 71.541 | 1 | 0.000 | 0.049 | |
| Step 4 | Single | 0.552 | 0.276 | 4.009 | 1 | 0.045 | 1.737 | 1.012-2.982 |
| | HCWs | | | 6.994 | 2 | 0.30 | | |

| | | | | | | | | |
|---|---|---|---|---|---|---|---|---|
| | Students | 0.827 | 0.356 | 5.402 | 1 | 0.02 | 2.287 | 1.138-4.594 |
| | Others | 0.060 | 0.308 | 0.038 | 1 | 0.845 | 1.062 | 0.581-1.942 |
| | Comorbidity | 1.558 | 0.367 | 18.040 | 1 | 0.000 | 4.750 | 2.314-9.749 |
| | Sleep latency | 0.535 | 0.113 | 22.550 | 1 | 0.000 | 1.708 | 1.370-2.131 |
| | Constant | -3.266 | 0.389 | 70.633 | 1 | 0.000 | 0.038 | |
| Step 5 | Single | 0.608 | 0.279 | 4.728 | 1 | 0.030 | 1.836 | 1.062-3.175 |
| | HCWs | | | 6.350 | 2 | 0.042 | | |
| | Students | 0.774 | 0.357 | 4.693 | 1 | 0.030 | 2.169 | 1.077-4.370 |
| | Others | 0.031 | 0.311 | 0.010 | 1 | 0.921 | 1.031 | 0.561-1.897 |
| | Comorbidity | 1.438 | 0.377 | 14.520 | 1 | 0.000 | 4.213 | 2.011-8.828 |
| | Family history | 0.915 | 0.462 | 3.924 | 1 | 0.048 | 2.497 | 1.010-6.176 |
| | Sleep latency | 0.499 | 0.115 | 18.803 | 1 | 0.000 | 1.646 | 1.314-2.063 |
| | Constant | -3.242 | 0.391 | 68.646 | 1 | 0.000 | 0.39 | |
