# Supplementary material for: Prevalence and Characteristics of Obsessive-Compulsive Disorder Among Urban Residents in Wuhan During the Stage of Regular Control of Coronavirus Disease-19 Epidemic
Source: Front Psychiatry. 2020 Dec 16;11:594167. doi: 10.3389/fpsyt.2020.594167 (PMC7772465; doi:10.3389/fpsyt.2020.594167)
Supplement: Supplementary file 3 [file Table_1.pdf]

Table 1 Univariate analysis of factors related to OCD

Item		Non-OCD N, ()%	OCD N, ()%	P	χ^2 or T（Z）
Total		444(82.07)	97(17.93)		
Gender	Male	190(82.61)	40(17.39)	0.726	0.123
	Female	254(81.67)	57(18.33)		
Age group(years), N	15-24	99(77.34)	29(22.66)	0.022	9.594
	25-34	192(79.34)	50(20.66)		
	35-44	88(88.89)	11(11.11)		
	≥45	65(90.28)	7(9.72)		
Monthly pay (RMB), N	<3000	140(79.10)	37(20.90)	0.442	1.635
	3000-5000	147(84.00	28(16.00)		

		)			
	>5000	157(83.07)	32(16.93)		
Marital status, N	Unmarried	180(75.95)	57(24.05)	0.001	10.738
	Married	264(86.84)	40(13.16)		
Education level, N	High school	97(85.09)	17(14.91)	0.194	4.719
	Junior college	104(90.64)	19(9.36)		
	Bachelor	161(82.56)	34(17.44)		
	Master degree	82(75.23)	27(24.77)		
Family members, N	1	28(84.85)	5(15.15)	0.320	3.508
	2-3	238(79.60)	61(20.40)		
	4-6	164(85.86)	27(14.14)		

	>6	14(77.78)	4(22.22)		
Employment status, N	Employed	293(84.68)	53(15.32)	0.020	9.797
	Retired	22(88.00)	3(12.00)		
	In school	87(72.50)	33(27.50)		
	Inoccupation	42(84.00)	8(16.00)		
Occupation, N	HCWs	123(85.42)	21(14.58)	0.002	12.357
	Students	80(70.80)	33(29.20)		
	Others	241(84.86)	43(15.14)		
District, N	Wuchang	187(82.02)	41(17.98)	0.487	5.451
	Qiaokou	24(80.00)	6(20.00)		
	Jiangan	24(80.00)	6(20.00)		
	Jianghan	48(87.27)	7(12.73)		
	Hongshan	103(78.03)	29(21.97)		
	Hanyang	37(84.09)	7(15.91)		
	Qinshan	21(95.45)	1(4.55)		

Duration of residence（month), N	0-6	85(84.16)	16(15.84)	0.182	4.869
	6-12	54(72.97)	20(27.03)		
	12-36	73(82.95)	15(17.05)		
	>36	232(83.45)	46(16.55)		
Exposure level, N	Low	368(82.88)	76(17.12)	0.311	2.336
	Medium	59(80.82)	14(19.18)		
	High	17(70.83)	7(29.17)		
Confirmed case, N	Yes	9(75.00)	3(25.00)	0.791	0.070
	No	435(82.23)	94(17.77)		
Suspected case, N	Yes	11(73.33)	4(26.67)	0.580	0.306

	No	433(82.32)	93(17.68)		
Asymptomatic case, N	Yes	13(65.00)	7(35.00)	0.043	4.113
	No	431(82.73)	90(17.27)		
Other mental disease, N	Yes	21(51.22)	20(48.78)	0.000	28.693
	No	423(84.60)	77(15.40)		
Family history, N	Yes	15(53.57)	13(46.43)	0.000	16.298
	No	429(83.63)	84(16.37)		
Subjective support score, mean ± SD		22.42±5.34	21.18±4.70	0.006	-2.755

Objective support score, mean ± SD		8.16±3.42	7.28±3.03	0.023	-2.281
Availability of support score, mean ± SD		7.07±2.06	6.87±2.08	0.192	-1.306
SSRS score, mean ± SD		37.66±8.73	35.32±7.39	0.002	-3.056
Sleep quality score, mean ± SD		1.92±0.72	2.22±0.82	0.001	-3.313
Sleep disturban		2.14±1.07	2.56±1.07	0.001	-3.474

ce score, mean ± SD					
Sleep latency score, mean ± SD		1.69±0.93	2.30±1.15	0.000	-5.179
Sleep duration score, mean ± SD		1.75±0.81	1.91±0.88	0.103	-1.631
PSQI score, mean ± SD		7.49±2.66	8.98±3.06	0.000	-4.374

RMB is China's currency, also know as yuan. HCWs refers to Health Care Workers. SSRS means Social Support Rating Scale. PSQI means Pittsburgh Sleep Quality Index.
